# Randomised controlled feasibility study of a school-based multi-level intervention to increase physical activity and decrease sedentary behaviour among vocational school students

**DOI:** 10.1186/s12966-017-0484-0

**Published:** 2017-03-21

**Authors:** Nelli Hankonen, Matti T. J. Heino, Sini-Tuuli Hynynen, Hanna Laine, Vera Araújo-Soares, Falko F. Sniehotta, Tommi Vasankari, Reijo Sund, Ari Haukkala

**Affiliations:** 1 Faculty of Social Sciences, University of Tampere, Kalevankatu 4, 33014 Tampere, Finland; 2 Faculty of Social Sciences, University of Helsinki, Helsinki, Finland; 3 University of Newcastle Upon Tyne, Newcastle upon Tyne, UK; 4 UKK Institute, Tampere, Finland; 5 Institute of Clinical Medicine, University of Eastern Finland, Kuopio, Finland

**Keywords:** Feasibility, Acceptability, Pilot trial, Self-determination theory, Self-regulation, Planning, Behaviour change technique use, Vocational upper secondary school

## Abstract

**Background:**

No school-based physical activity (PA) interventions among older adolescents have demonstrated long-term effectiveness, and few of them so far have addressed sedentary behaviour (SB). Based on behavioural theories and evidence, we designed a multi-level intervention to increase PA and decrease SB among vocational school students. This study investigates feasibility and acceptability of two main intervention components and research procedures. We also examine uptake of behaviour change techniques (BCTs) by the participants.

**Methods:**

Design was an outcome assessor blinded, cluster-randomised controlled trial. Four classes of students (matched pairs) were randomised into one intervention and one control arm. The intervention consisted of (1) a 6-h group-based intervention for students, (2) two 2-h training workshops to reduce their students’ sitting in class for teachers, and (3) provision of light PA equipment in classrooms. At baseline (T1), mid-intervention (T2) at 3 weeks, post-intervention (T3) and 6 months after baseline (T4) we measured hypothesised psychosocial mediators and self-reported PA and sitting. Objective assessment of PA and SB (7-day accelerometry) was conducted at T1, T3 and T4. Body composition (bioimpedance) was measured at T1 and T4. Students and teachers in the intervention arm filled in acceptability questionnaires at T3.

**Results:**

Recruitment rate was 64% (students) and 88.9% (teachers), and at T3, all post-intervention measurements were completed by 33 students (retention 76.7%) and 15 teachers (retention 93.8%). Acceptability ratings of sessions were high (students *M* = 6.29, scale 1–7), and data collection procedures were feasible. Intervention arm students reported increased use of BCTs, but uptake of some key BCTs was suboptimal. BCT use correlated highly with objective measures of PA. Based on both self-report and student evaluation, teachers in the intervention arm increased the use of sitting reduction strategies at post-intervention and T4 follow-up (*p* < .05).

**Conclusions:**

We detected willingness of the target groups to participate, good response rates to questionnaires, adequate retention, as well as acceptability of the trial protocol. Investigation of BCT use among students helped further enhance intervention procedures to promote BCT use. After making necessary modifications identified, intervention effectiveness can next be tested in a definitive trial.

**Trial registration:**

ISRCTN34534846. Registered 23 May 2014. Retrospectively registered.

**Electronic supplementary material:**

The online version of this article (doi:10.1186/s12966-017-0484-0) contains supplementary material, which is available to authorized users.

## Background

Adolescents engage in far less physical activity (PA) than recommended [1] and excessive sedentary behaviour (SB). Lack of PA and excessive SB are more prevalent among those with lower socioeconomic status, for example lower educational level, i.e. vocational students, compared to high school students [2, 3]. Socioeconomic health disparities [3] call for targeted interventions.

Schools are a promising setting for increasing youth PA, due to high reach. Given the declines in activity occurring during adolescence, surprisingly few studies have evaluated school-based interventions targeting PA and SB among older adolescents: As few as ten RCTs have been reported [4], most of which had methodological aspects not meeting Cochrane criteria for low risk of bias. Six interventions were effective short-term, and none of the few studies evaluating long-term effectiveness evaluation, were effective. To our knowledge, none of these interventions were pilot tested in comprehensive feasibility studies.

In other age groups, successful interventions include multiple components and target many levels of the school system with an exclusive focus on PA, and do not use classroom-based education alone (e.g. [5–7]). Use of theory in intervention design has been associated with better outcomes in school-based health promotion [6, 7]. Several theories have successfully explained youth PA behaviours, including those focusing on strength of motivation [8], quality of motivation (Self-Determination Theory, SDT) [9] and effective self-regulation of behaviour [10, 11]. Such theories imply a range of potentially effective Behaviour Change Techniques (BCTs) [12] such as prompting self-monitoring of behaviour and coping planning.

A limitation of much intervention research has been a lack of investigation of fidelity on the part of the participants. Even in interventions delivered with high fidelity, only a minority of participants may take up the intended BCTs, [13]. If participants do not understand the skills taught to them, nor enact them in their daily life, the intervention may fail to have effects [14]. Thus, assessing BCT use has recently been identified as a key focus for process evaluation [15]. Some earlier work has indeed measured goal setting or action planning as a result of an intervention e.g. [16–18], but rarely the whole range of BCTs that the participants are expected to enact (see e.g. [13, 14]). One further gap in previous research has been a focus on self-regulatory strategies at the expense of strategies that individuals may use to maintain optimal motivation for the targeted behaviour. In this study, we aimed to evaluate a wide range of BCTs, including “self-motivational strategies”, which individuals can use to help themselves to better engage in the process of behaviour change, to gain understanding for the intervention optimisation.

A phased, iterative approach [19] was used to design and pilot a complex multi-level intervention to address PA and SB of students, including six group sessions delivered directly to students, as well as teacher training and changes in physical choice architecture to reduce SB in classrooms. The intervention aimed to increase PA especially among those with low or moderate levels of PA. To improve odds of success of a definitive trial, this study was conducted to examine the feasibility and acceptability of research procedures and intervention content. As lower educated populations may respond to health promotion less favourably than the higher educated (e.g.[20]), feasibility testing of interventions for target group may be particularly critical.

We aimed to assess the feasibility of the *Let’s Move It* trial protocol and its procedures for the recruitment, consent, assessment, allocation, intervention, and retention procedures, amongst students and teachers in vocational schools. More specifically, the aims were 1) to determine the feasibility of conducting a definitive RCT and 2) to evaluate acceptability of the intervention and trial procedures; assess: a) acceptability of recruitment, randomisation and consent procedures, b) acceptability and feasibility of collecting reliable and valid data on outcomes (acceptability of measurement instruments), c) acceptability of the two main intervention components, d) enactment of BCTs prompted by the student intervention, and examine this by baseline PA level, as an indicator of the acceptability among the main target group.

As a secondary research question, we investigated the changes in PA and SB (students) and student sitting reduction activities (teachers). The findings are expected to enable the optimisation of the intervention and research procedures of a later cluster-RCT. We hypothesise that the recruitment, intervention, measurement and trial procedures will be feasible and acceptable, thus allowing us to proceed with a full RCT for effectiveness evaluation.

## Methods

### Feasibility trial design

We conducted an exploratory feasibility study (ISRCTN34534846) in a vocational school unit in Southern Finland. The study was an outcome assessor blinded, cluster-randomised controlled trial with the class as the unit of randomisation. The intervention was a multi-level intervention, delivered to both teachers as well as students directly. All participants were blind to allocation at baseline.

With the term vocational school, we refer to organisations providing vocational education and training, as opposed to those providing the general upper secondary education (“high schools”). In Finland, selection into either vocational or general upper secondary education occurs after universal basic education, typically at 15 years-of-age. The participating school provides training in several fields, including technology and transport, business and administration, as well as health and social services.

### Randomisation

In the collaborating school, four classes attending a compulsory health education course in the last study period of spring 2014 were enrolled. The classes were first paired using the available background information (size and gender composition) and then a statistician used a computerised random number generator to assign classes into control and intervention arms.

### Participants

**Fig. 1:**
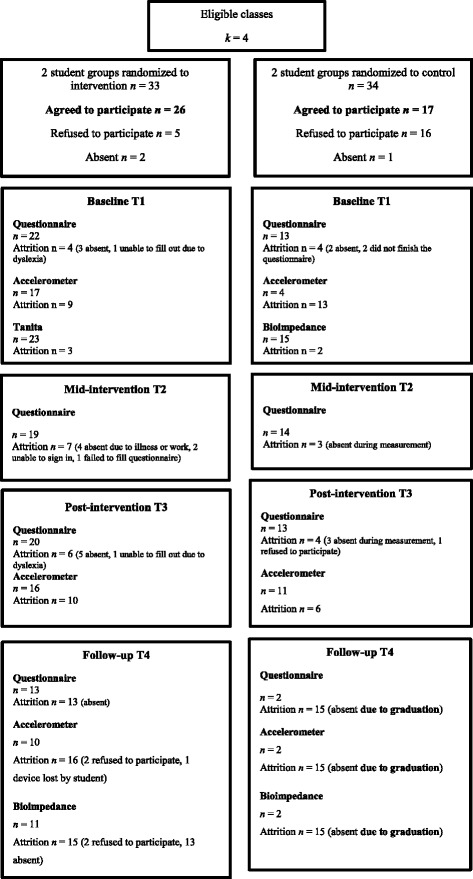
Flow diagram (student participants)

We included all vocational students in the class as participants, and we invited the teachers that taught these student groups in that teaching period and whose classes involve a large amount of sitting to participate. Exclusion criteria included insufficient knowledge of the Finnish language, and any medical conditions preventing participants from engagement in PA. Exclusion criteria for teachers included teaching in classrooms (workshops) which do not involve sitting. All classes that teachers identified to involve students sitting most of the time (e.g. mathematics) were targeted (for more information on the development of the teacher intervention, see [21]). Sample size target was defined by practical and resource considerations as 60 students and 20 teachers, and was deemed adequate for the study’s purpose. As we aimed to assess feasibility and acceptability, not effectiveness in terms of changes in outcomes, we did not conduct power calculations. For participant flow diagrams, see Fig. 1 (students) and Additional file 1: Figure S2 (teachers).

#### Student recruitment

A researcher presented the study to each class in April 2014. The students were given 2 days to consider participation, and in another session consent forms were delivered for consideration. Students gave their informed consent blind to allocation. Consenting students were requested to fill in the baseline questionnaire and to take part in bioimpedance measurements in the same session. Students who declined consent were given alternative tasks related to health education (e.g. task on lactose intolerance/nutrition).

#### Teacher recruitment

Core and vocational class teachers, teaching the classes during that teaching period (*n* = 18), were invited. Of them, 16 came to the information session. Everyone consented and were requested to fill in the baseline questionnaire. After this, the random allocation of the arms was concealed. Workshop I for took place after this.

### Interventions

The intervention included both individual and environmental level changes. The objective of the intervention was defined as increasing total PA of the students, more specifically, to 1) increase *moderate-to-vigorous physical activity* (MVPA), 2) decrease *proportion of sedentary behavior (SB,* sitting and lying), and 3) increase *interruptions of SB*. The two latter objectives were mainly addressed by altering choice architecture by creating environmental *opportunities* to be more physically active in school. All three objectives were addressed in a six-session group intervention for students. The main intervention components were 1) a group intervention for students, and 2) workshops for teachers. Additionally, 3) physical choice architecture was altered by providing PA equipment to enable light PA in classrooms (see Additional file 1: Figure S1).

Intervention components are briefly described next (see Additional file 2: Table S1 and Additional file 3: Table S2 for a more detailed description). Additional file 1: Table S3 describes study timeline.

#### Intervention development

Theory, evidence synthesis and stakeholder input informed the stepwise intervention development [22]. The intervention was based on original research in the target population, a systematic review of 10 relevant RCTs [23], and behaviour change theories (see [22]), mainly SDT and self-regulation theories. We drew on specific theories, relevant to youth PA, to base intervention activities on theoretical insights on how to foster autonomous, self-determined motivation [9] and effective self-regulation of behaviour [10, 11], following principles of motivational interviewing [24] that provides practical strategies in line with the SDT.

Our participatory approach involved stakeholders (school staff, students) in development. After assessing needs and strengths of the target group and setting, specifying intervention objectives, identifying key theoretical determinants and mediators, we selected BCTs to change the determinants. Programme components and materials were pre-tested among users [22]. Both BCTs delivered by the facilitators and those that rely on active individual enactment by participants (e.g. goal setting, action planning) were defined. Additional file 1: Figure S1 displays an intervention overview and Additional file 2: Table S1 & Additional file 3: Table S2 display session aims, content, BCTs delivered, and materials. Facilitators were female and had a university degree in social psychology.

#### Student intervention

A member of the researcher team delivered six group sessions, using engaging teaching methods with individual and small group tasks. BCTs followed a logical pattern with a discussion of the motivations, beliefs, barriers and facilitators related to PA in participants’ lives, then goal setting and action planning in later sessions, followed by the introduction of subsequent BCTs on a weekly basis concluding with coping planning (problem solving) in the end, adapting tested exercises (e.g., volitional help sheet [25]). Autonomy supportive interaction aimed to foster autonomous motivation for PA [9, 24]. Table 1 shows examples of intervention activities. Support materials were both printed sheets as well as a LifeGuide website, with essentially the same content as in the sessions, guiding students through e.g. SMART goal setting. Two short booster sessions, via email and/or by phone, were given, following principles of motivational interviewing [24]. Boosters were also delivered in social media.

**Table 1:** Examples of intervention activities in the Let’s Move It student intervention

Objectives	Activities	BCTs	Determinants
Identifying Personal Motives Group Activity (Session 3)			
Students: • engage in reflecting personally meaningful reasons to be physically active • learn about the various positive consequences of PA • link physical activity to their current wellbeing concerns and values • understand that their peers have positive attitudes toward PA and appreciate its consequences • engage in “change talk”, arguments in favour of PA • understand that it is possible to enhance/develop PA motivation	• Cards showing beneficial consequences of PA are on table • Students are asked to select at least one benefit card, reflecting on personally important reasons for being physically active (or why they would like to increase their PA) • Students show the benefit card they have chosen and tell others why • Facilitator leads discussions so that students speak of a range of positive consequences of PA (especially consequences not related to extrinsic goals, e.g. appearance) • Facilitator highlights that everyone has their own personally meaningful reasons to be physically active (e.g. not everyone has to be motivated by competition) • If students are reluctant to select a card or talk about it, this is accepted.	5.1. Information about health consequences 5.2. Salience of consequences 5.3. Information about social and environmental consequences 5.4. Information about emotional consequences 6.3. Information about others’ approval 13.2 Framing/Reframing	Knowledge Outcome expectations Autonomous motivation (integrated regulation) Descriptive norm Self-efficacy
Coping Plan Consultants (Session 5)			
Students • learn strategies to identify and overcome barriers to PA • are introduced to the term ‘coping planning’ and understand its relevance • learn various strategies to overcome PA barriers, e.g. how to restructure social and physical environments to support achievement of PA goals • understand that it is possible to tackle various barriers and obstacles in youth PA, and increase their self-efficacy • when “coaching” the imaginary person, students are able to both take the role of an “outside expert”, and focus on solutions rather than problems, thus enhancing their self-efficacy	• In groups of four, students read an imaginary case of an adolescent: a description of barriers in his/her life that make PA difficult • Small groups identify the PA barriers and try to generate solutions • Students are encouraged to draw from experiences from their own lives, if they want to • Groups present their case and solutions to the whole class • Facilitator emphasizes problem solving (instead of only identifying the barriers) and normalizes having various barriers in youth’s life	1.2. Problem solving 4.2. Information about antecedents of behaviour 3.1. Social support 13.1 Identification of self as role model	Behavioral self-regulation Self-efficacy
Fitness Knowledge Quiz (Session 6)			
Students understand • that physical fitness (PF) consists of three different dimensions (strength, mobility and aerobic fitness) • types of PA that improve dimensions of PF, and that some ways of exercising can improve all three (e.g. gymnastics) while others focus more on other areas (e.g. yoga on mobility & strength, or running on aerobic fitness) • how often they should exercise according to national recommendations, at the same time highlighting that even small increases provide benefits • the importance of increasing PA gradually • and can identify how PF is related to personally important outcomes (e.g. strength and back pain)	• In a playful quiz, students answer questions related to physical fitness/PA in groups of five (1st round: what kind of sports enhance the different dimensions of physical fitness, 2nd round: How often adolescents should exercise according to the national PA recommendations?) • Groups write down their answers • Facilitator highlights that even little movement is better than nothing • PE teacher attended the session and acted as an impartial referee • Facilitator highlights how different dimensions of PF should be trained equally and one should always take one’s starting level into consideration (rules for safe training) • Facilitator highlights importance of finding the type of PA each one enjoys and the linkage with personal motives, preferences and values	4.1. Instruction on how to perform a behavior 5.1. Information about health consequences 5.2. Salience of consequences 8.7. Graded tasks 15.1. Verbal persuasion about capability	Knowledge Outcome expectations Autonomous motivation Self-efficacy

***Control treatment*** was standard care, i.e., normal curriculum delivered in the school, plus a leaflet on recommendations for youth PA after completing T1 questionnaires.

#### Teacher intervention

In *two face-to-face group workshops* teachers were motivated and trained to use different strategies to reduce students sitting during class. Teachers were given *access to a webpage* with practical examples on how to integrate sitting interruption in teaching and a *booklet* aiming at increasing motivation to reduce students sitting and at supporting rehearsal of different strategies. The booklet targeted teachers’ perceived benefits of sitting reduction and disadvantages of uninterrupted sitting (information about consequences of the behavior) with the aim of increasing motivation to change. Throughout the materials, the sitting reduction strategies were presented in light of their positive consequences (Strength, Relaxation, Energy, Flexibility), to enhance salience of consequences of the behaviour. This was followed by instructions on how to perform the behavior (i.e. presenting sitting reduction strategies to use during class, such as the use of activity break guidance cards, links to online videos guiding activity breaks, and written instructions on e.g. pedagogical methods to reduce sitting (e.g. learning café). The booklet also had an interactive exercise where teachers were encouraged to set a goal, for a specific teaching period, and to plan where and how to use sitting reduction strategies with student groups. In addition, it contained practical tips on how to motivate students to reduce sitting in classes. This same content was available on the website (see Additional file 4: Supplementary material for sample pages). *Equipment* was provided for classrooms. Workshop II prompted teachers to plan how to overcome barriers and provided further sitting reduction strategies. Teachers had a chance for further personalised support from the facilitator via email and phone. The intervention was informed by formative research in another sample of teachers [21].

#### Teacher control arm (written information)

Teacher participants in the control arm received a leaflet informing them about the benefits of interrupting sitting.

### Measurements and blinding

Data were collected from students at pre-intervention baseline (Time 1, T1), during intervention at 3 weeks (T2), after the intensive intervention at 4–6 weeks (T3) and 6 months after baseline (T4). In addition to participant blinding at recruitment, outcome assessors (assessing accelerometry and bioimpedance) were blinded. At follow-ups, assessors requested participants not to reveal the arm.

### Primary outcome measures

Primary outcome was acceptability and feasibility of procedures for recruitment, measurement, retention, and for the intervention. Recruitment rates were measured as ratio of invited participants consenting/eligible. We aimed to assess acceptability of allocation procedures by examining reasons for drop-out in discontinuing participants and comparing attrition rates between arms. Suitability of measurement procedures were evaluated based on completion rates. Attrition rates were established as discontinuation of intervention and loss to follow-up.

Among students, intervention acceptability was assessed using two items: “I would recommend participating in this program to other vocational students”, with response alternatives ranging from 1 (completely disagree) to 7 (completely agree), and ”How much did you like the programme as a whole?”, with response alternatives ranging from 1 (Not at all) to 5 (Liked very much). Each session was rated: “How satisfied were you with today’s programme?“(response alternatives 1–7). Anonymous feedback questionnaires ensured that students were aware that their responses could not be linked to their names.

Among teachers, two questions measured intervention acceptability: First, “Evaluate your overall satisfaction with the workshop on a scale 0 (=not at all satisfied) to 100 (very satisfied). My overall satisfaction is __”, and second, “Please evaluate the pilot study of sitting reduction, and answer the question: I would recommend participation in the study to my colleagues“ (response alternatives 1 = Completely disagree, 5 = Completely agree).

Some BCTs do not require a high level of engagement (e.g. provide information on health consequences), but others require active participant engagement (e.g., prompt self-monitoring) to have effects. The use of the latter type of BCTs (i.e., actively enacted) was measured by self-report (16 self-enacted BCTs). The item stem “During the last 2 weeks, have you done the following?” was followed by each BCT (see Table 4 for the items). We developed the measure together with BCT experts, adapting similar measures [13]. While some BCTs may be “one-off”, the effectiveness of some BCTs requires regular or frequent repetition (e.g., self-monitoring [26]). Therefore, the response options were for four BCTs: Not at all true (1) – True (5), and for twelve frequency-dependent BCTs between 1 and 5 (1 = not once, 2 = about once in 2 weeks, 3 = about 1–2 times per week, 4 = about every second day, 5 = daily). In addition to self-regulatory BCTs, we included measures of “self-motivational BCTs” that individuals may use to maintain optimal motivation.

A dichotomous variable was created with a category for *non-compliers* consisting of respondents choosing “not once” or “about once in 2 weeks”, and a *weekly complier* category consisting of an aggregation of respondents choosing “about 1–2 times per week”, “about every second day”, and “daily”.

### Secondary outcome measures

#### Students

In this feasibility study, secondary outcomes are objectively measured and self-reported PA and SB, body composition (muscle and fat mass), self-reported screen time, well-being, and self-reported use of BCTs. The primary outcomes of the future RCT, i.e. *objective measures* of PA and SB are here investigated as secondary outcomes (measured at T1, T3, T4). MVPA, sedentary time, and standing-ups were based on accelerometry [27]. The 3-axis Hookie AM 20-accelerometer (Traxmeet Ltd, Finland) is a valid measurement tool among adults [27] and youth [28].

The accelerometer attached to a flexible belt which the participants were instructed to wear around their hip for 7 days during waking hours, except during shower and other water activities. The accelerometers collected the tri-axial data in raw mode in actual g-units. The data was analyzed in 6 s’ epoch length. PA was categorised into three intensity categories based on metabolic equivalents (MET): light (1.5 − 2.9 MET), moderate (3.0–5.9 MET) and vigorous (more than 6 MET) [29]. Time spent in sitting and reclining positions were combined to indicate SB, time standing still was analysed separately [30]. It is possible to accurately determine whether the participant is standing, sitting or lying by applying the tri-axial information from the accelerometer. Since the body position during walking is upright and the direction of Earth’s gravity vector is constant, the vertical position (angle) of the accelerometer can be identified during walking. This known position can be then used for recognising different body postures. In standardised conditions, standing can be separated from sitting and lying with 100% accuracy, and sitting from lying with 95% accuracy (24). Daily amount of stand-ups (breaks in sedentary time) was calculated on the basis of the number of lying/sitting periods ending with a standing up. A PA diary indicated non-wear time. At T1 and T3, research assistants delivered accelerometers to participants, and collected them and the diaries in the subsequent week at the school. For some students, the school term ended before the 7-day accelerometry finished, hence, they were given pre-paid envelopes to return device and the diary. At T4, the accelerometers were delivered and collected by research staff at schools.

At T1 and T4, research assistants measured students’ body composition and height. We used bioelectric impedance analysis technology to measure body composition (Tanita MC-780MA, Tokyo, Japan). The bioimpedance measure relied on a proprietary algorithm that was not developed for this study population. Inaccuracy can thus be expected, and although the inaccuracies should impact the intervention and control groups similarly, the extent of bias remains unknown.

Upon entering the room where the measurement took place, the participants were instructed to remove as much clothing as they were comfortable with in order to increase the accuracy of the measurement. They were then instructed to step on the electrodes on the base (two electrodes per foot) and wait for the research assistant’s signal before instructed to hold the handles on the bioimpedance measurement device. The device then measured their weight, and during this, the research assistant demonstrated how the participant should proceed to hold the handles in appropriate degree angle from their body, grabbing firmly with their hands without tensing the arm muscles.

*Self-report measures* (T1–T4) included socio-demographic background factors, behaviour, and self-reported health. Participants completed the online questionnaires in the school at T1, T2 and T3. At T4, if in-school data collection was impossible to arrange, an email invitation to the questionnaire was sent, with two reminder emails.

*Website usage* was measured by sign-in counts on the LifeGuide platform.

#### Teachers

*Self-report measures* (T1–T4). Activities to reduce student sitting during the previous 2 weeks were measured with seven items: a) Informing students they are allowed to walk freely in class; b) proposing students to stretch independently; c) having an activity break (led by the teacher, a student or an on-screen video); d) using teaching methods that include moving (e.g. learning cafe); e) having a break in class, and; f) another strategy. The responses ranged from 1 to 6 (1 = Never, 2 = Less frequently than once a week, 3 = Approximately once a week, 4 = Approximately 2–3- times a week, 5 = Approximately once a day and 6 = More often than once a day).

Student perceptions of teacher sitting reduction activities were measured with the statement “Most of my teachers…” followed by seven items: 1) inform students of favourable consequences of sitting reduction, 2) offer possibilities to reduce sitting time, 3) give permission to move freely in the classroom during class, 4) propose to students being independently physically active during class, 5) use activity breaks, 6) organise teaching activities that include movement and 7) inform students about adverse consequences of prolonged sitting. The response scale ranged from 1 to 6 (1 = never, 2 = once a month or more seldom, 3 = couple of times per month, 4 = about once a week, 5 = a few times a week, 6 = daily).

### Statistical analyses

BCT use was analysed in two ways: Average use of BCTs as well as proportion of weekly users of the key BCTs. The changes in the two BCT use sumscores were investigated with repeated-measures ANOVA. Correlations of BCTs with objectively measured PA were investigated to explore criterion validity.

Accelerometer data were analysed for all days the device was worn for over 8 h. All available data were included for questionnaire and bioimpedance measurement. Student t-tests were used to assess baseline differences in psychosocial variables and BCT use. Repeated-measures ANOVA was used to evaluate the interaction between group (intervention versus control) and time (baseline, follow-up) for the primary and secondary outcome measures. Analyses were conducted using SPSS version 22.0.

Clustering of data was not accounted for in the analyses, as the study was not designed for adequate power to detect changes in the variables. All analyses used *k* = 2 clusters for intervention and *k* = 2 clusters for control group.

## Results

### Recruitment

Figure 1 shows the CONSORT flowchart for this study. In the four eligible classes of students, out of 67 eligible individuals 64 were reached and invited to participate in the study. Altogether 43 (67.2%) of the reached 64 adolescents provided signed informed consent. In one of the groups, two opinion leaders voiced concerns and dissent when filling out the consent forms. This behaviour had a great impact in changing the class intentions of project participation and influenced the general atmosphere in class so that several participants withdrew their participation, resulting in as few as five consenting participants from this class (see Table 2 for recruitment by class).

**Table 2:** Recruitment and participants by class

	Total students	Filled in T1 questionnaire	Recruitment rate (%)^a^
Intervention class A	15	10	67%
Intervention class B	18	16	89%
Control class C	21	5	24%
Control class D	13	12	92%
Total	67	43	64.2%

^a^Calculated based on all students (*n* = 67), although 64 were reached for invitations

Teachers’ flow chart is shown in Additional file 1: Figure S2. Out of 18 teachers, 16 gave their consent to participate.

### Baseline characteristics of control and intervention arms

The baseline questionnaire was filled by 40 students, of which 35 completed it. Table 3 shows baseline characteristics. The total sample was on average 18.9 years (SD = 1.67) (range 17–25), 85% (*n* = 34) female, BMI of 23.02/m^2^ (*SD* =7.01), engaged in MVPA 46.56 (*SD* = 14.95) min/day and were sedentary for 68% (*SD* = 7) of the day.

**Table 3:** Baseline characteristics of the student sample. Numbers indicate mean (SD)

	Total	Control^a^	Intervention^b^
BMI	23.02 (3.94)	21.51 (3.66)	24.00 (3.88)
Fat %	25.95 (7.01)	23.63 (6.89)	27.47 (6.81)
Fat free %^c^	74.05 (7.01)	76.38 (6.89)	72.55 (6.81)
Muscle %	70.30 (6.67)	72.50 (6.54)	68.87 (6.49)
Inactivity min	563.32 (79.34)	535.86 (72.09)	569.78 (81.62)
Inactivity %	68 (7)	64 (7)	69 (7)
MVPA min	46.56 (14.95)	48.13 (12.50)	46.19 (15.79)
MVPA %	6 (2)	6 (1)	6 (2)
Stand-ups per day	25.37 (7.80)	28.56 (7.43)	24.62 (7.91)

^a^Sample size: bioimpedance *n* = 15, accelerometer *n* = 4

^b^Sample size: bioimpedance *n* = 23, accelerometer *n* = 17

^c^Fat % and Fat free % do not always add to 100% due to rounding

We did not detect differences between participants allocated to the control arm and those allocated to the intervention arm in sex, age, BMI, MVPA, inactive time or the number of standups (*p* > 0.05 for all differences).

### Intervention procedures and attendance

#### Students

Of the 26 intervention participants, everyone attended at least 1 session. There was no one who did not complete the intervention. Most of the participants also attended the majority of intervention sessions (attendance in Additional file 1: Table S4); the mean number of attendees was 18.3 (SD = 5.4, Md = 20.5). Eight out of 24 (33.3%) participants responded to the booster phone call. If not reached via phone, they were sent an email. All intervention class members were contacted and invited to a Facebook group, 22 responded to the invitation message, and 17 (70.8%) joined the Facebook Let’s Move It group. According to Lifeguide session logs, no users logged in to browse the intervention contents apart from during the sessions held at the school.

#### Teachers

Of the allocated eight teachers, seven (88%) participated in at least one workshop. Seven teachers attended the workshop I and six the workshop II. One teacher received individual guidance from the research team, due to being unable to attend any of the workshop II training sessions. The only person not continuing the study was in the intervention arm.

### Acceptability of intervention components

#### Students

After the intensive phase of the intervention, at T3, participants reported high levels of satisfaction with intervention (*M* = 6.29 on a scale of 1–7, *SD* = 0.56, *n* = 21) and reported being willing to recommend the intervention to peers (*M* = 4.60 on a scale of 1–5 *SD* = 0.60, *n* = 20). None of the participants responded lower than five on the first question or three on the second, indicating high acceptability of intervention content.

#### Teachers

Teachers reported high satisfaction with the workshops, with mean ratings averaging near 90 out of 100 (Workshop I: *M* = 89.18, *SD* = 7.36; Workshop II: *M* = 89.83, *SD* = 5.31), indicating high acceptability. After the workshops, teachers reported high willingness to recommend the intervention to colleagues (*M* = 4.83 on a scale of 1–5 *SD* = 0.41, *n* = 6).

### Uptake of behaviour change techniques

Additional file 1: Table S5 shows that the use of BCTs correlated with objectively measured PA (e.g., *r* = .57, *p* = .011), indicating criterion validity. Intervention arm reported increased use of BCTs post-intervention (*p* < .05 for both sumscores) (Table 4). BCT use at T3 was not correlated with baseline PA. Mean scores and use of each BCT by study arm is shown in Table 4.

**Table 4:** Changes in self-reported BCT use from pre-intervention (T1) to post-intervention (T3) by group. Numbers indicate mean (SD)

Measure	Group	T1	T3
BCTs sumscore (for general BCTs)	Control	2.9 (0.9)	2.6 (1.5)
	Intervention	2.6 (1.0)	3.3 (1.0)
BCT sumscore (for frequency-dependent BCTs)	Control	2.6 (0.6)	2.1 (0.7)
	Intervention	2.5 (0.8)	2.8 (1.1)
I have set PA goals for myself (“*goal setting, behaviour*” 1.1)	Control	3.8 (1.1)	3.5 (1.6)
	Intervention	3.1 (1.3)	3.8 (1.1)
I have made a detailed plan to carry out PA (“*action planning*” 1.4)	Control	3.0 (1.6)	2.7 (1.7)
	Intervention	2.6 (1.3)	3.3 (1.2)
I have written down my plan (in e.g. my calendar) (“*action planning*”, 1.4)	Control	2.4 (1.6)	2.0 (1.6)
	Intervention	2.2 (1.4)	2.9 (1.3)
I have divided large PA goals into smaller goals (“*graded tasks”,* 8.7)	Control	1.3 (0.7)	1.6 (1.0)
	Intervention	2.0 (1.1)	2.7 (1.3)
I have thought about what positive consequences regular PA would bring into my life (“*keeping in mind/reminding oneself of positive consequences of PA*”^a^)	Control	3.8 (1.1)	3.0 (1.2)
	Intervention	3.1 (1.4)	3.3 (1.3)
I have thought about my PA goals (“*thinking about own goals*”^a^)	Control	3.9 (1.0)	3.1 (1.3)
	Intervention	3.3 (1.5)	3.4 (1.2)
I have monitored my own PA, e.g. by logging bouts of PA in my PA diary or mobile app (“*self-monitoring of behaviour*” 2.3)	Control	2.1 (1.5)	1.7 (1.1)
	Intervention	1.9 (1.3)	2.4 (1.4)
I have compared my actual PA with the PA goal that I had set (“discrepancy between current behaviour and goal”, 1.6)	Control	2.2 (1.3)	1.8 (1.0)
	Intervention	2.4 (1.3)	2.6 (1.1)
If I have not reached my PA goal, I have considered what went wrong (“*barrier identification a)*”^a^)	Control	2.1 (1.3)	2.1 (1.1)
	Intervention	2.2 (1.4)	2.6 (1.6)
I have thought about what reasons for PA are important for me personally (“*thinking about /reminding oneself of one’s own motives*”^a^)	Control	3.5 (1.2)	2.7 (1.6)
	Intervention	3.1 (1.2)	3.2 (1.2)
I have considered what kind of situations prevent me from realizing my PA plan (“*barrier identification b)*”^a^)	Control	2.9 (1.4)	2.4 (1.5)
	Intervention	2.6 (1.1)	2.8 (1.0)
I have planned ways to overcome barriers to being physically active (“*problem solving*”, 1.2)	Control	2.3 (1.2)	1.7 (0.9)
	Intervention	2.7 (1.2)	2.9 (1.3)
I have tried out new ways of being physically active (“*behavioural experiments*”, 4.4)	Control	2.3 (1.3)	1.8 (0.6)
	Intervention	1.9 (0.9)	2.3 (1.2)
I have asked my family or friends to be physically active with me (“*obtaining social support*”, 3.1)	Control	2.3 (1.3)	1.9 (1.3)
	Intervention	2.4 (1.0)	2.8 (1.2)

*Note*. In all BCTs, Control *n* = 10 Intervention *n* = 18, except question “If I have not reached my PA goal I have considered what went wrong”, where intervention *n* = 17 (participant indicated having reached their PA goal). The corresponding BCT is specified in parenthesis (numbering refers to BCT Taxonomy v1, Michie et al., 2013,)

^a^ = Novel BCT for this study

Table 5 shows the proportions of those reporting (at least) weekly use of each frequency-dependent BCTs. In the intervention arm, BCTs use ranged from 32 to 80%. The BCTs related to self-regulation (e.g., self-monitoring, coping planning) were less popular than BCTs related to motivation (e.g., thinking about own motives for PA). However, comparing the difference between proportion of BCT users across intervention and control arm, it is apparent that in fact *self-monitoring*, *graded tas*ks, and *barrier identificatio*n were the BCTs that the intervention arm reported using more, and the motivational BCTs (*thinking about positive consequences of PA*, *thinking about own motives*) and *discrepancy between current behaviour and goal* were in turn very frequently reported by the controls also.

Facilitator faced difficulty in getting students engaged in home assignments (“The Leisure-time Dares”), possibly because vocational school students were not used to homework from school in other subjects. The facilitator endeavoured to underline the personal relevance of completing the exercises. Some students would lose sheets (e.g. with planning and goal setting tasks) delivered to them in sessions.

**Table 5:** Percentages of participants reporting at least weekly compliance with BCTs at T3

	Overall % (*N* = 32–33)	Control % (*n* = 13)	Intervention % (*n* = 19–20)
Graded tasks	36	15	50
Keeping in mind positive consequences of PA	73	69	75
Thinking about one’s goals	76	69	80
Self-monitoring of PA	39	23	50
Discrepancy between current behaviour and goal	48	38	55
Problem solving	34	38	32
Thinking about one’s own motives	67	62	70
Barrier identification b)	58	46	65
Behavioural experiments	55	38	65
Obtaining social support	33	23	40

### Completion of questionnaires and measurements, drop-out

#### Students

Thirty five students completed full T1 questionnaire. Questionnaire at T2 and T3 was completed by 33 and at T4 by 13 students. This translates to 51% total retention (intervention: 50%; control 53%). Venn diagrams clarify proportions of participants completing various parts of measurements (Additional file 1: Figure S3). On average across T1, T3 and T4, 73% of distributed accelerometers were worn for a minimum of 4 days, at least 8 h per day, indicating adequate but not optimal adherence to objective PA measurement.

In the intervention arm, 26 students consented, but T1 questionnaire was filled by 22 as three students were ill during that day and one’s dyslexia hindered completion on time. Most non-completion was due to students being absent from schools on the measurement days, not active refusal to participate (see Fig. 1). Active refusal reasons ranged from being ill to being too busy.

At T3, one control participant actively asked to discontinue the study, but no-one in the intervention arm. A drop-out analysis (t-tests) did not reveal differences in T3 respondents and others in sex, age, study year or working status (e. g., part-time job), and they were also alike in MVPA, sedentary time and BMI at T1.

By T4, one class had graduated and could not be reached. The T4 drop-out rate including the graduated class is 65.1% and excluding is 51.6% (i.e., based on the three classes still in school). All T4 measurements were completed by two control and ten intervention participants.

#### Teachers

Out of 18 eligible teachers, 16 attended the study information event and 100% gave consent to participate. Only two teachers in the intervention arm dropped out, one by T3, and another one at T4.

### Changes in secondary outcomes

Changes in the outcome variables for the definitive trial were small. Since there was a lack of participants that fully completed the measurements, all trends must be interpreted with caution.

#### Students

Table 6 shows changes in objectively measured and self-reported behaviour. No changes were significant over time between arms, as may be expected in a Phase II study, not sufficiently powered to detect changes in outcomes. No major effects can be inferred. The accelerometry measurement was compromised due to human error by a research assistant during data collection (correct charging of the accelerometer devices), further lowering the sample size.

**Table 6:** Changes in secondary outcome measures. Numbers indicate mean (SD)

Measure	Group (*n*)	T1	T3
Proportion of MVPA^a^	Control (3)	0.06 (0.01)	0.08 (0.06)
	Intervention (12)	0.06 (0.02)	0.07 (0.03)
Proportion of passive time^a^	Control (3)	0.66 (0.06)	0.63 (0.14)
	Intervention (12)	0.68 (0.07)	0.66 (0.10)
Standing ups	Control (3)	30.48 (7.79)	26.03 (10.78)
	Intervention (12)	26.62 (7.48)	26.12 (13.51)
Self-reported PA	Control (10)	2.7 (1.8)	2.3 (1.7)
	Intervention (18)	3.4 (2.1)	2.8 (2.1)
Self-reported sitting, weekdays	Control (10)	294.0 (177.1)	360.0 (155.6)
	Intervention (18)	320.0 (162.4)	383.3 (155.9)
Self-reported sitting, weekend	Control (10)	294.0 (137.0)	324.0 (158.0)
	Intervention (18)	331.7 (127.9)	385.0 (143.4)
Self-reported breaks in sitting	Control (10)	2.0 (0.7)	2.3 (0.6)
	Intervention (18)	2.3 (0.7)	2.4 (0.6)
Self-reported breaks in sitting at school	Control (10)	1.4 (0.5)	1.6 (0.7)
	Intervention (18)	2.2 (1.0)	2.4 (0.8)

^a^Out of total wear time

#### Teachers

Teachers’ sitting reduction activities were significantly different between arms post-intervention according to both student evaluations, F(1,26) = 8.50 (*p* = 0.007, partial *η* ^*2*^ 0.246) and self-reported assessments F(1, 13) = 16.26 (*p* = 0.001, partial η^2^ 0.556). This indicates a large effect size (Additional file 1: Figures S4 and S5). The teachers’ own and students’ estimates correspond to each other: Both indicate teachers used the various strategies approximately once per week on average in the intervention arm, and approximately once a month in the control arm.

Table 7 summarises identified problems and solutions to potentially improve research procedures. Intervention content improvements are reported elsewhere [22].

**Table 7:** Problems detected in research procedures and solutions generated for the main trial

	Problem identified	Solution generated for the definitive RCT
1	For the control group, as a standard treatment, we gave the participants PA and sitting reduction health education brochures after the baseline measurements ended. The control participants did not find this practice pleasurable or sensible.	Change the control group to be a “no-treatment control”, i.e. refrain from giving any additional brochures to participants.
2	Questionnaire burden. The questionnaires were perceived as too lengthy and boring by the participants.	Decrease length and number of questionnaires: 1) No T2 questionnaire for controls. 2) No questionnaires after each session for intervention participants. 3) Decrease number of questions in all questionnaires.
3	Accelerometers were not returned quickly or at all in pre-paid envelopes. Vacation periods interfered with return.	Research assistants aim to collect accelerometers directly from schools in person. Measurements not scheduled too close to vacation period.
4	Several students’ non-participation was due to being ill or other reason for not attending school on the day(s) when research team were in school to collect data.	Reserve enough days to return to schools for bioimpedance and accelerometer measurements, schedule several days for same class in order to maximize participation.
5	The strategies in which students were motivated and instructed to wear the accelerometer were too scarce (in research assistant – participant face-to-face session). Even slight changes from T3 to T4 instructions were related to increase in the days worn.	Improved instructions for RCT: For example, we instructed the participant to immediately put on the accelerometer (instead of just giving it to them), and simplified the self-report log associated with the accelerometer. We also added motivational content to the accelerometer instruction script.
6	Several students cited “not remembering” as the reason for not wearing the accelerometer.	SMS reminders to help participants remember to put on accelerometer in the mornings in the RCT.
7	In recruitment of one class, initial reception of research was positive, but in the session where consent was to be signed and questionnaire filled, negative group norms arose perhaps due to 1) different researchers were present for recruiting and subsequently hosting survey measurement or 2) lag between recruitment and survey.	1) Avoid changes in personnel per class. Script even more carefully the recruitment session to avoid recruitment bias (to ensure similar recruitment between classes/recruiters). 2) Ask consent and provide survey in the same session as recruitment occurs. Filling in baseline survey right after recruitment and study info.
8	Difficulty in reaching third year students at follow-up (graduation).	1) Include only first or second year students in RCT. 2) Shorten the originally intended 24-month-follow-up into 14 months, to avoid the realistic risk of not locating participants for the last follow-up.
9	M (SD) of the outcome variables.	Power calculations accordingly (we used these data to inform a power calculation for sample size of the RCT).
10	Recruitment rate was 67.2% and drop-out after intervention (T3) in intervention arm and control arm 23.1% and 23.5%. Accelerometer drop-out due to human error by research assistant in preparing the accelerometer devices.	1) Adjust target recruitment rates accordingly. 2) Improve procedures to prevent study drop-out: E.g.: training of interpersonal and communication skills among data collection staff, improve motivation for participating in accelerometer study, add SMS reminders for accelerometer wear time, improve staff skill in preparing accelerometers.
11	Intervention timing was suboptimal, starting at the middle of the fourth (last) period of the school year.	1) As intervention effects need to be investigated in different seasons (PA seasonal effects), intervention start cannot be timed at the beginning of the school year for all of the RCT batches. Instead, ensure that intervention activities start promptly after each period starts. 2) Teacher activities to be placed in the beginning of school years.

## Discussion

We tested feasibility and acceptability of a school-based intervention to increase adolescent PA and to reduce their SB. We found support for the hypothesis that the intervention components and research procedures were feasible and acceptable, but also identified several needs for improvement. We did not find evidence that allocation procedures would not be found acceptable. Only few participants explicitly provided reasons for dropout, and the original aim of investigating and comparing these across arms could not be followed up on. The main intervention components were highly rated by both teachers and students. No adverse events were detected.

We compared some main results to prior similar studies [4], of which 7 out of 10 reported recruitment rates. The average recruitment rate was 93.3% (range 60.0–96.9%). The nine studies that reported an attrition rate [4] had on average a rate of 31.2% (range 0.0–54.0%) for post-intervention and 27.9% (2.6–34.3%) for follow-up. Our study fell short of these points of comparison, which is partially explained by higher measurement burden (more extensive objective measurement compared to any of the prior trials: inclusion of both 7-day accelerometry and bioimpedance): Indeed, questionnaire participation was high in all our measurement points (see Additional file 1: Figure S3). Only two prior trials included objective PA measurement, hence, few relevant comparisons exist. Also, this study population is from a lower socioeconomic position, known to have generally lower research participation. On the other hand, drop-out was not due to active refusals (except for one) but rather not reaching the students during measurement days, and one class graduating before T4. Questionnaire completion rates were high. Several identified improvement needs allow us to enhance both recruitment and retention before the full RCT (Table 7). In sum, these parameters were considered to be acceptable and indicate that, after modifications, these procedures are viable for a full RCT.

Visits to the website were few, in line with another school-based intervention [31] whose website the youth did not frequently visit. Degree of exposure and engagement with optionally usable websites may be difficult to enhance, thus interventions should not uncritically use digital tools if they cannot ensure usage of those tools at leisure time. We decided to decrease emphasis on the website for the full trial.

Despite positive feedback on the intervention, the uptake of the intended BCTs among students was moderate. An earlier study that had investigated the use of all protocol-defined BCTs had not assessed BCT use in the control arm [13]. Contrasting intervention and control arm BCT use against each other showed, that whereas the “motivational BCTs” were reported to be used at least weekly by the majority of the participants in both arms, self-regulatory BCTs were less often reported by the controls. The hypothesised key BCTs, self-monitoring, coping planning and graded tasks (e.g. [4]), were more used by intervention participants. High ratings of the motivational BCTs in both arms may imply several issues. Firstly, as they only involved “thinking” or “bringing to mind” one’s motives, using motivational BCTs is easier than the use of BCTs that require an active and more effortful engagement (e.g.,coping planning). Second, the items may have been too ambiguous to respond to. Third, simply thinking about one’s own motives and values may not be effective in supporting behaviour change: Future research needs to better conceptualise what effective motivational self-management entails.

The intervention aim to target especially those with low to moderate PA, was achieved: BCT use was unrelated to baseline PA, indicating that BCTs were not taken up by only those already high in PA. It should be noted that evaluation of BCT use is only relevant for “cognitive” interventions where BCTs are expected to be actively enacted, as opposed to interventions targeting “automatic” processing or environmental opportunities. Indeed, we did not evaluate sitting reduction BCTs uptake among students, as sitting reduction was an environmental intervention for students.

Fidelity assessments have been often restricted to aspects of intervention delivery (e.g., [32]). A recent study reported a minority of participants enacting all of the prompted BCTs [13]. Hence, a failure to find an effect may not only be a failure of the program content or an inadequate delivery, but also failure of the target group to use the intended BCTs. If feasibility studies identify which BCTs are the least used, then interventions could better be optimised to either a) better prompt the less frequently used BCTs, or b) consider modifying the intervention content. We optimised the intervention to enhance uptake of BCTs, by e. g. emphasising benefits and ease of use, providing helpful examples and models, and adding reminders (see examples of added activities to enhance BCT use in Table 8).

**Table 8:** Examples of added activities to enhance BCT use

• Provide better rationale for BCT use both in sessions and in campaign materials in cafeterias
• Provide more concrete examples of goals & plans for students, e.g. cafeteria campaign showcasing examples of these key BCTs
• Make self-monitoring optional for some homework tasks: Instead of requesting students to self-monitor their PA daily after each session, for the optimised intervention, compulsory requests for daily monitoring over 7 days will be done only after sessions 1 and 5
• Increase time for discussion of how to obtain social support over the sessions
• Decrease program focus on website and instead focus BCT use on readily available student workbooks

Limitations include a focus on one school only, but resources did not allow for a larger study, and in fact, key intervention components act at the individual and interpersonal rather than the school level. Second, due to the novelty, the self-report measure for BCTs was not robustly validated. However, criterion validity is indicated by the relatively high correlations with objectively measured PA. Furthermore, it was more precise than previously reported enactment scales using dichotomous responses only (e.g. [13]), measuring also the use of BCTs in control participants. Third, the process may have benefited from a priori set criteria for indicators of acceptability and feasibility. It should be noted that the measurement of body composition using bioimpedance assessment entails reliability problems. For example, validated, population-specific FFM prediction equation should be applied, which was not done here. Thus one should not place too much emphasis on the bioelectric impedance analysis results, and particularly not interpret them against e. g. population values, that were measured using different algorithms. Finally, as all classes resided in the same school unit, contamination may pose a risk – however, as students were from different educational tracks, they are unlikely to interact with each other, and furthermore, that would likely have low influence the primary outcomes of feasibility and acceptability of this study. However, to reduce the risk of contamination, randomisation is carried out by school in the definitive RCT.

Implications for practice are many: Parameter estimates of PA and SB outcome measures, and recruitment and completion rates informed the RCT power calculations and the RCT design. Testing the feasibility and rehearsing the standard operational procedures allowed appropriate revisions prior to a definitive RCT, with insights useful for designers of similar trials. The usefulness of rehearsing the procedures was evident in that due to one research team’s mistake, the accelerometers were not correctly charged. This led the team to devise more stringent and better specified standard operational procedures (SOPs) for the full RCT. The same was true for low recruitment success of one of the classes, which led to improvements of our procedures and thus increased acceptability of recruitment procedures.

## Conclusions

This study provided evidence that the Let’s Move It intervention can be implemented in the vocational school setting, and enabled optimization of procedures. Our novel way of conceptualizing of acceptability and feasibility via the use of a BCT enactment questionnaire may prove a useful methodological tool for further feasibility studies, as it reveals which BCTs are used the most and least, thus identifying weak spots. Paying systematic attention to BCT enactment in process evaluation may improve effectiveness of future interventions. The Let’s Move It intervention has been designed to be disseminable nationwide, but prior to that, the effectiveness of the finalised intervention should be established in a full-scale trial.

## Additional files

Additional file 1: Figure S1. Overview of the intervention tested in the feasibility study. **Figure S2.** Teacher flow diagram. **Table S3.** Study timeline: Data collection and intervention activities by week (numbered). **Table S4.** Ratings of each session by students in anonymous feedback forms. **Table S5.** Pearson’s correlations between use of frequency-dependent BCTs and moderate-to-vigorous activity (MVPA). **Figure S3.** Venn diagrams for students’ questionnaire completion (at T1-T3), illustrating various combinations of participation and non-participation. **Figure S4.** Student evaluations of teacher activities to reduce student sitting. **Figure S5.** Teachers’ self-reported activities to reduce sitting. (PDF 735 kb)

Additional file 2: Table S1. Specific content of the face-to-face intervention module for students. (PDF 365 kb)

Additional file 3: Table S2. Specific content of teacher intervention module. (PDF 330 kb)

Additional file 4: Supplementary material. (PDF 202 kb)

## Abbreviations

BCT: Behaviour Change Technique
MET: Metabolic equivalent
PA: Physical activity
RCT: Randomised controlled trial
SB: Sedentary behaviour
