# Supplementary material for: Randomised controlled feasibility study of a school-based multi-level intervention to increase physical activity and decrease sedentary behaviour among vocational school students
Source: Int J Behav Nutr Phys Act. 2017 Mar 21;14:37. doi: 10.1186/s12966-017-0484-0 (PMC5361824; doi:10.1186/s12966-017-0484-0)

Supplementary materials

Additional Figure 1. Overview of the intervention tested in the feasibility study.

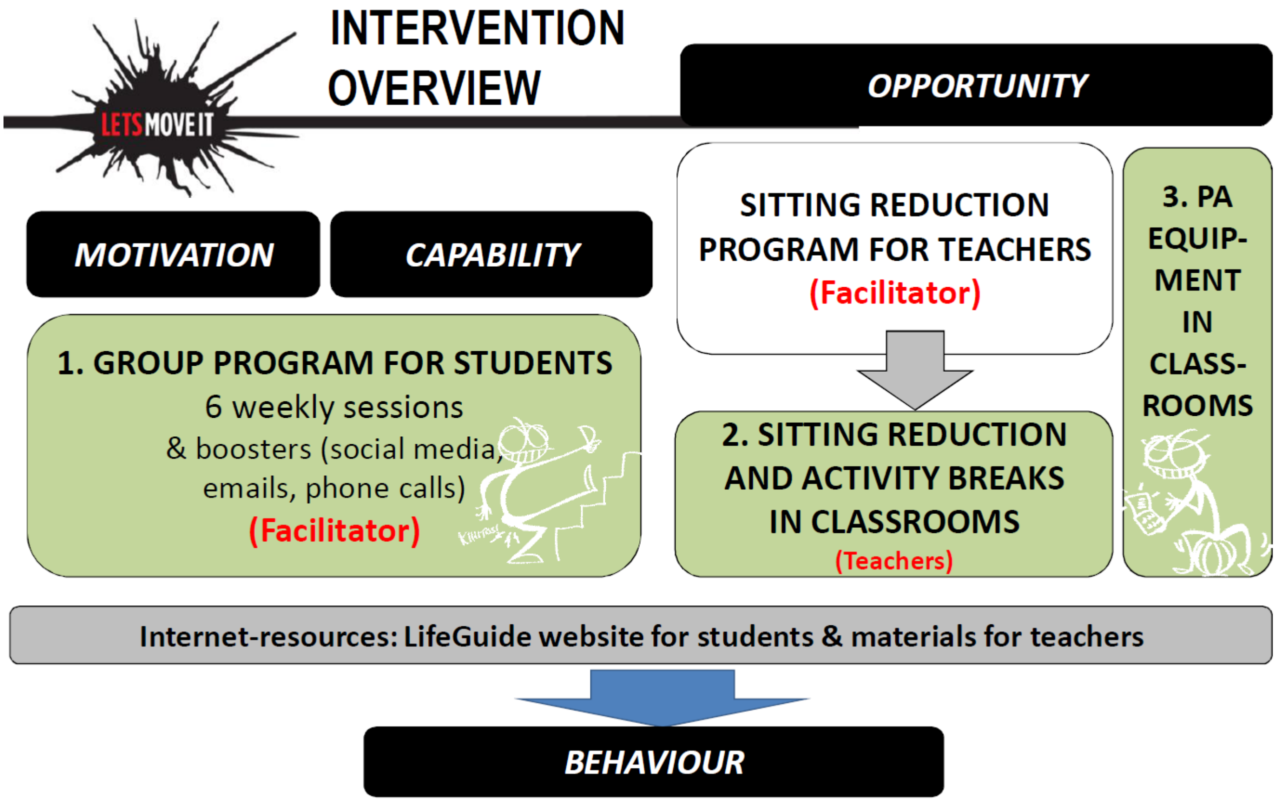

Additional Figure 2. Teacher flow diagram.

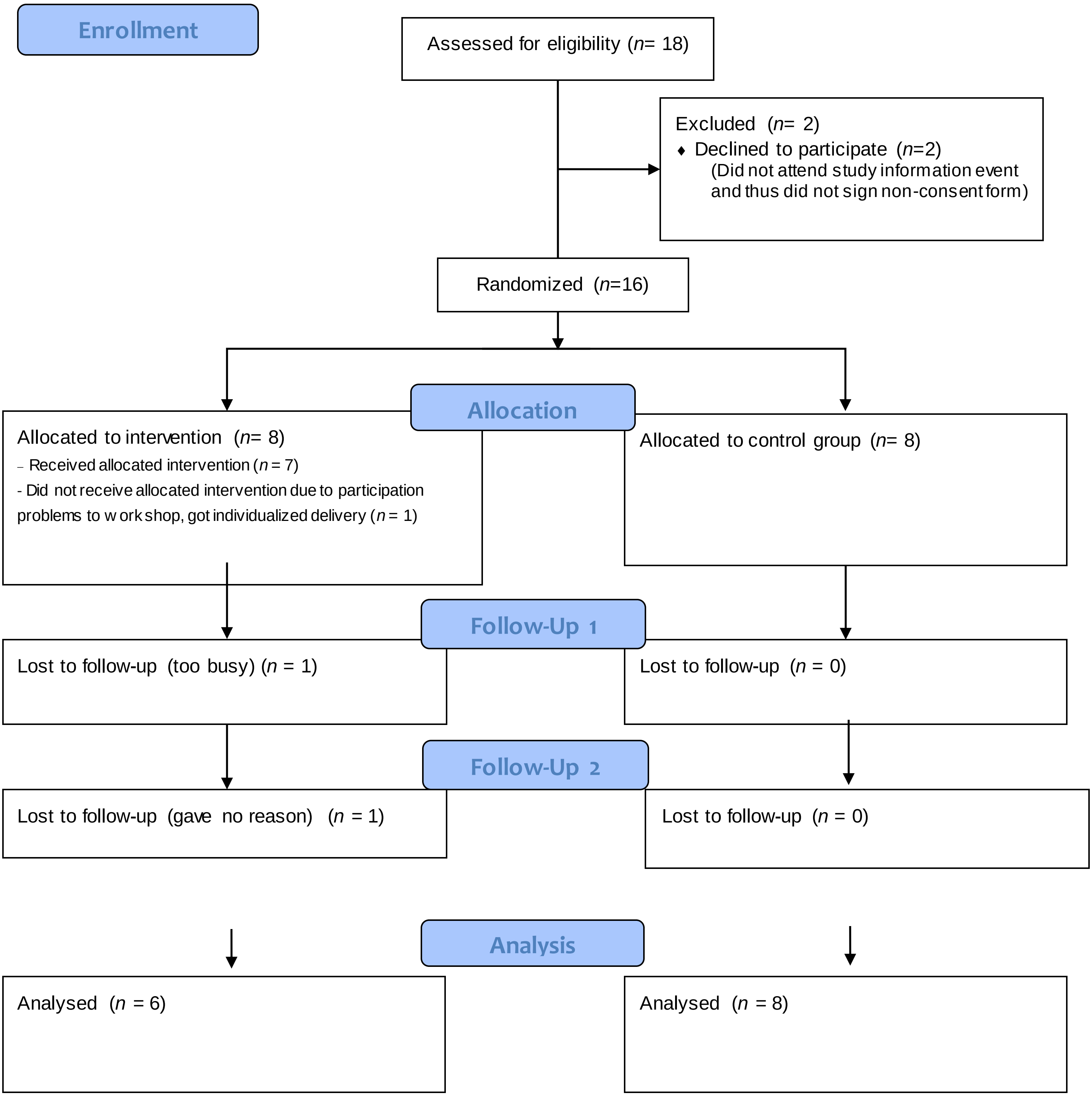

Additional Table 3. Study timeline: Data collection and intervention activities by week (numbered).

Week	0	1	2	3	4	5	14	20	- 24
Data collection									
Baseline measurement	x								
Mid-intervention measurement				x					
Post-intervention measurement						x			
Follow-up measurement									x
Intervention activities									
(1) GROUP PROGRAM FOR STUDENTS									
Group face-to-face sessions (1, 2) *		x	x	x	x	x			
Booster sessions (phone, or if unreached, email) * (1, 2)							x		
Internet resources (LifeGuide)		x	x	x	x	x	x	x	
Social media group (1, 2) *							x		
(2) SITTING REDUCTION									
Sitting reduction workshops for teachers		x		x					
Students' sitting reduction in classrooms *			x	x					
(3) PA EQUIPMENT IN CLASSROOMS (CHOICE ARCHITECTURE)									
PA equipment in classes			x	x	x	x	x		

Additional Table 4. Ratings of each session by students in anonymous feedback forms.

Session	Mean rating (SD)	*n*
1	6.35	17
2	6.20	20
3	6.23	22
4	6.26	23
5	6.14	7*
6/5-6	6.24	21*
All sessions	6.24 (0.07)	

Note: Range 1-7, with higher numbers indicating greater satisfaction.

*Note. Sessions 5 and 6 were delivered in the same day for one of the intervention classes, hence, they only filled in one session evaluation form for the sessions 5-6.

Additional Table 5. Pearson's correlations between use of frequency-dependent BCTs and moderate-to-vigorous activity (MVPA).

		MVPA T1	MVPA T3	BCTs T1	BCTs T3
MVPA T1	Pearson's r	—	0.710 **	**0.570** *	-0.247
	p-value	—	0.003	0.011	0.340
	Upper 95% CI	—	0.896	0.814	0.265
	Lower 95% CI	—	0.310	0.157	-0.650
MVPA T3	Pearson's r		—	0.475 *	**0.132**
	p-value		—	0.030	0.538
	Upper 95% CI		—	0.752	0.509
	Lower 95% CI		—	0.054	-0.286
BCTs T1	Pearson's r			—	0.550 **
	p-value			—	0.002
	Upper 95% CI			—	0.766
	Lower 95% CI			—	0.222

* p < .05, ** p < .01, *** p < .001

Additional Figure 3. Venn diagrams for students' questionnaire completion (at T1-T3), illustrating various combinations of participation and non-participation.

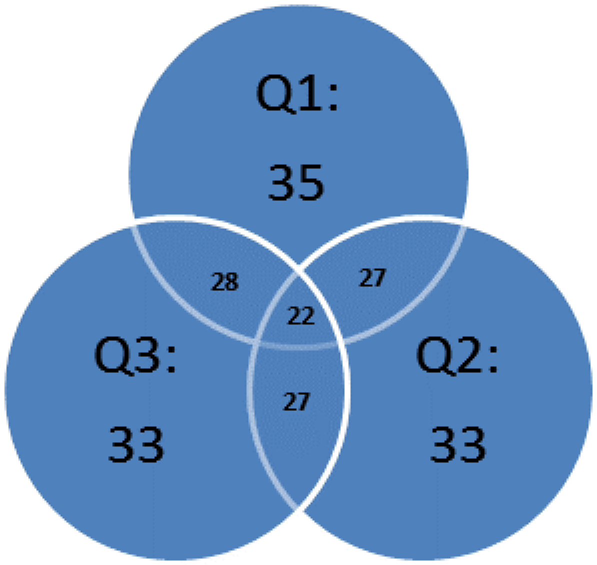

Note. Of the 35 students (13 control vs. 22 intervention) who completed T1 questionnaire, 27 completed T2 questionnaire (11 vs. 16) and 28 completed T3 questionnaire (10 vs. 18). Of the 33 people who completed T2 questionnaire (14 vs. 19), 27 completed T3 questionnaire (12 vs. 15), and all three questionnaires were completed by 22 people (9 vs. 13). Thus full data was acquired from 22 participants (9 control vs. 13 intervention), whereas partial data was obtained from 43 people (17 vs. 26).

Additional Figure 4.

Student evaluations of teacher activities to reduce student sitting. Mean of 7 questions. Error bars represent 95% confidence intervals.

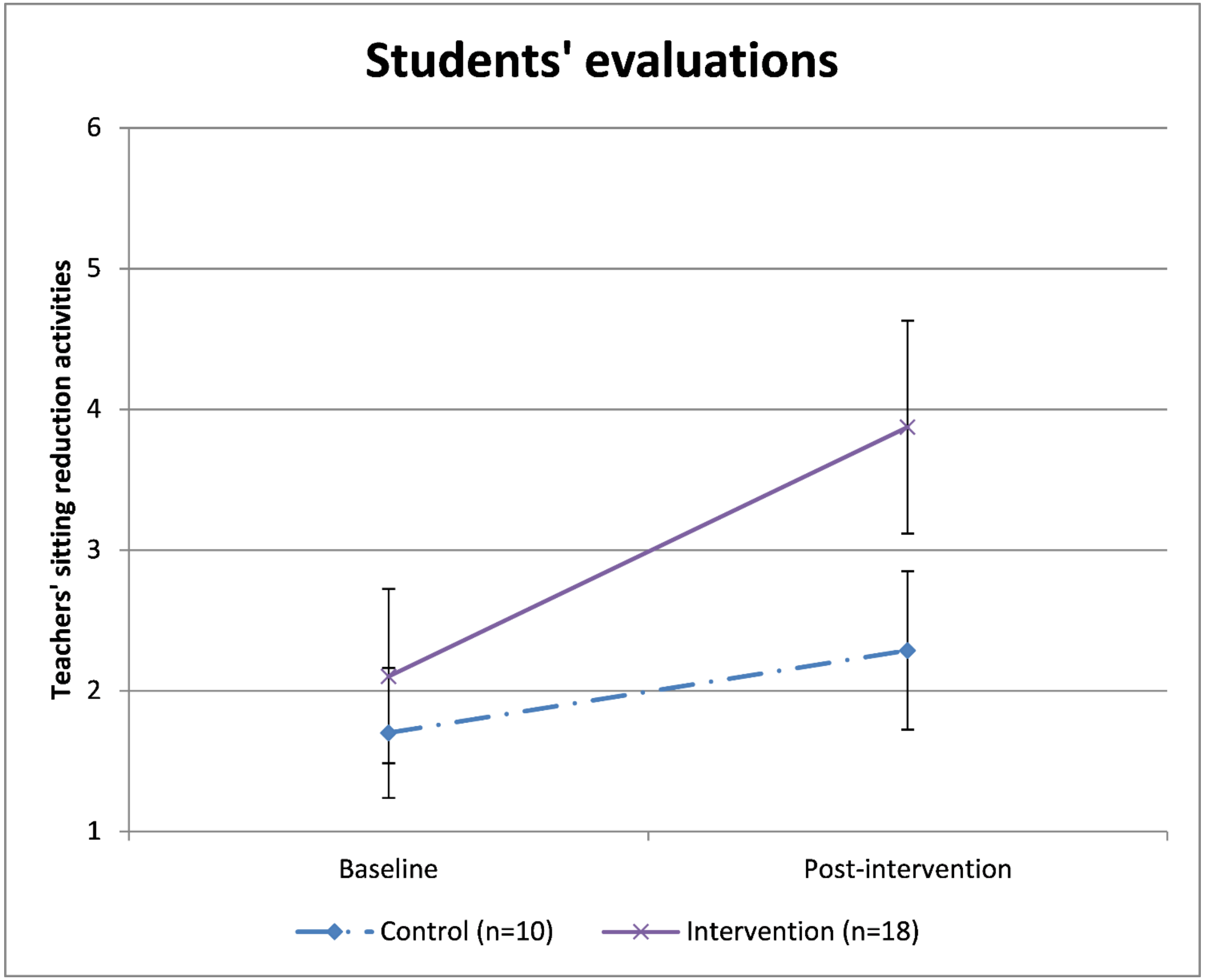

Additional Figure 5.

Teachers' self-reported activities to reduce sitting. Mean of 8 questions. Error bars represent 95% confidence intervals.

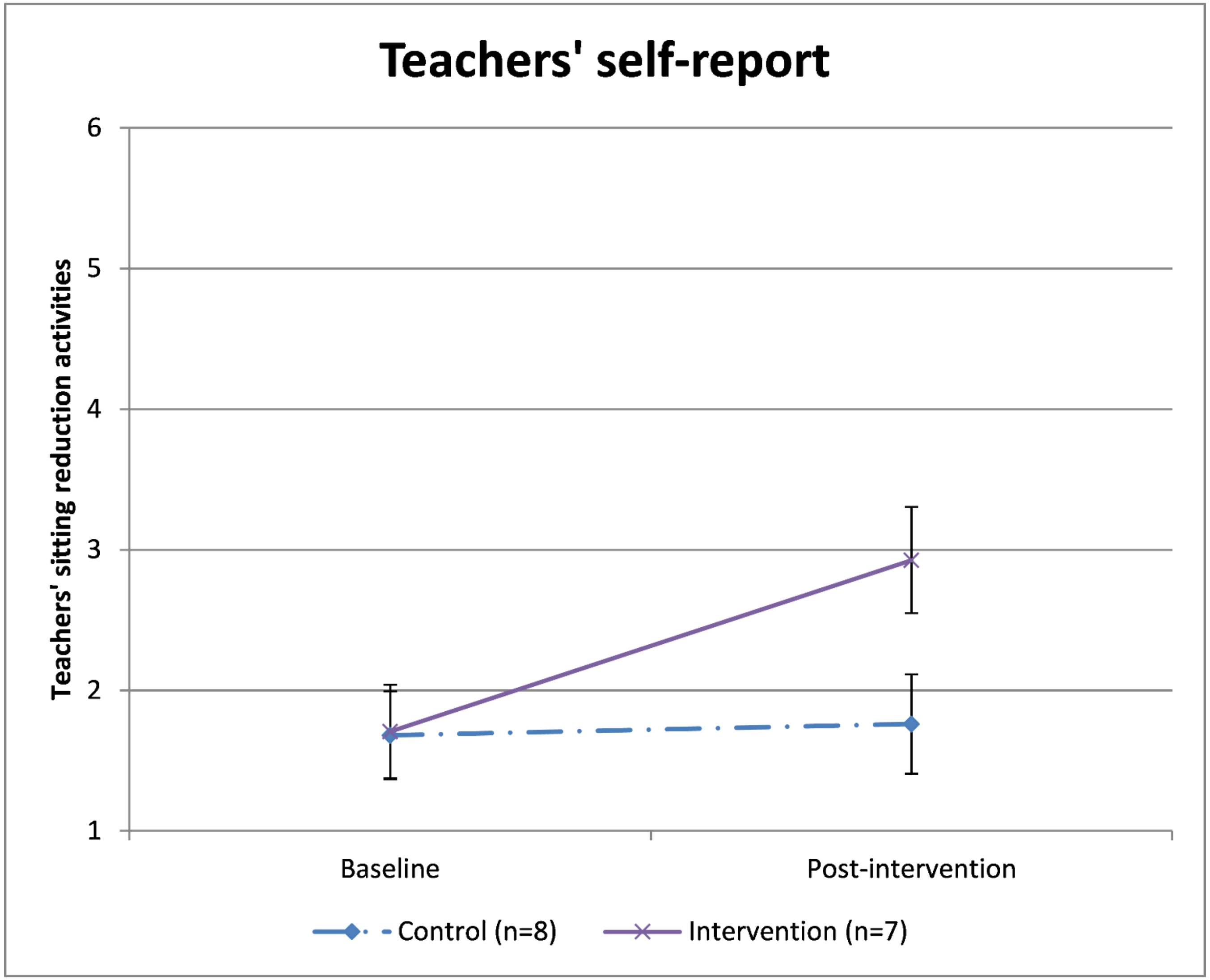

Supplement: Supplementary file 1 — Overview of the intervention tested in the feasibility study. Figure S2. Teacher flow diagram. Table S3. Study timeline: Data collection and intervention activities by week (numbered). Table S4. Ratings of each session by students in anonymous feedback forms. Table S5. Pearson’s correlations between use of frequency-dependent BCTs and moderate-to-vigorous activity (MVPA). Figure S3. Venn diagrams for students’ questionnaire completion (at T1-T3), illustrating various combinations of participation and non-participation. Figure S4. Student evaluations of teacher activities to reduce student sitting. Figure S5. Teachers’ self-reported activities to reduce sitting. (PDF 735 kb) [file 12966_2017_484_MOESM1_ESM.pdf]
