# Supplementary material for: Randomised controlled feasibility study of a school-based multi-level intervention to increase physical activity and decrease sedentary behaviour among vocational school students
Source: Int J Behav Nutr Phys Act. 2017 Mar 21;14:37. doi: 10.1186/s12966-017-0484-0 (PMC5361824; doi:10.1186/s12966-017-0484-0)
Supplement: Supplementary file 2 — Specific content of the face-to-face intervention module for students. (PDF 365 kb) [file 12966_2017_484_MOESM2_ESM.pdf]

Additional Table 1. Specific content of the face-to-face intervention module for students.

Intervention session	Aims	Activity description	Key Behaviour Change Techniques (BCTs)	Mediator (theory-based construct)	Materials	Mode of delivery
Session 1: Kick-off	1) Provide rationale of the intervention, 2) Create a safe working environment, 3) Address and discuss consequences of PA and SB.	In the beginning of the session a group formation activity was done using "mood cards". In this exercise everyone got to introduce themselves and tell something about their day to get to know each other. Group rules were discussed and settled on. Students were provided with an outline of the intervention program. Consequences of PA and SB and descriptive norms related to these behaviors were addressed by presenting students with opening statements related to the behaviours that they subsequently discussed and made arguments either for or against. In the end of the session, students were assigned with homework on the project website further addressing the themes discussed in class.	Social support (unspecified)<br>Framing/Reframing<br>Information about social and environmental / emotional / health consequences<br>Salience of consequences<br>Information about others' approval	Outcome expectations (also SB)<br>Descriptive norm (also SB)<br>Autonomous motivation<br>Positive group climate	Mood cards<br>Powerpoint presentation:<br>Group rule sheet &<br>Consequences of PA and SB discussion points	Face-to-face in class<br>Individual home assignment on the project website
Session 2: Why would I be physically active	1) Students link physical activity to their current well-being and related concerns, 2) Students experience the instant positive consequences of PA, 3) Students learn to self-monitor physical activity, 4) Students learn about the consequences of excessive sedentary time and how to reverse these	This session started with revising last week's learnings, the group rules, and home assignments. This was followed by a short introduction and group conversation about sitting: 1) when and where does one spend sedentary time during the day, 2) what are the consequences of being excessively sedentary, 3) how to break extended sedentary periods and what are its the positive effects. This mini lecture and group discussion was followed by a short, playful activity break. Before and after the break students rated how they were feeling. They were encouraged to observe how the	Salience of consequences<br>Information about social and environmental / health / emotional consequences<br>Monitoring of emotional consequences<br>Demonstration of the behavior	Outcome expectations (also SB)<br>Descriptive norm (also SB)<br>Autonomous motivation (integrated motivational regulation)<br>Knowledge (SB)	Powerpoint presentation:<br>Summary of session 1<br>Group rule sheet<br>Mini lecture on SB<br>Work sheets for well-being mapping	Face-to-face in class<br>Individual home assignment sheets

		short burst of PA affected their mood, focus, & energy. After the activity break, students were asked to individually assess how satisfied they are with different areas of their well-being (mood, sleep / rest, time management, personal relationships, physical activity, studies & work, self-esteem, nutrition). Following the individual rating students were encouraged to discuss with a class mate, how increasing physical activity might affect these areas. This was followed by a group summary of the conversations. Finally, as a home assignment, students were encouraged to keep a PA diary for 7 days and to record how they feel after being / not being physically active. Students were suggested to use prompts to remind them to fill in the diary.	Behavioral practice/rehearsal<br>Generalization of a target behavior<br>Graded tasks<br>Information about others' approval<br>Self-monitoring of behavior<br>Instruction on how to perform the behavior<br>Comparative imagining of future outcomes<br>Verbal persuasion about capability<br>Social support practical (SB)	Self-efficacy (also SB)<br>Positive group climate	Equipment for the activity break<br>Mood rating spread sheets<br>PA diary sheets for home assignment	
Session 3: How and where could I be physically active?	1) Students get and accurate idea of their current level of PA, 2) Students learn about different PA opportunities, 3) Students find personally meaningful ways to be physically active, 4) Students perceive a positive social norm regarding PA	The session started with summarising last week's discussion about the consequences of PA and SB, and then reviewing with a class mate the PA diary home assignment. Students were encouraged to give positive feedback to each other. After the class mate discussions a short activity break was held. This was followed by a group exercise where the students got to select cards presenting positive consequences of PA. Each student picked the card that presented the consequences they found most meaningful. Choices were discussed in a standing circle among the whole group. Next, student were asked to draw a map of different PA	Information about social and environmental / emotional / health consequences<br>Feedback on behavior / Social reward<br>Social support (unspecified)<br>Demonstration of the behavior<br>Behavioral practice/rehearsal	Outcome expectations (also SB)<br>Autonomous motivation (integrated motivational regulation)<br>Descriptive norm<br>Self-efficacy<br>Behavioral self-regulation	Powerpoint presentation: Summary of session 2<br>Cards on consequences of PA<br>Work sheets for the PA opportunity map exercise	Face-to-face in class<br>Individual home assignment sheets<br>Project website provided ideas for PA experiment execution

		opportunities within their community. They were divided into small groups and each group focused on one specific environment. The groups' task was to come up with as many interesting physical activities as possible that can be done in this environment. The findings were discussed as a whole group. This week's home assignment was to experiment with one new type of PA. The students were encouraged to select the trial activity from the opportunity map they had created. Finding workout partners was also encouraged. Each student was handed a worksheet where they could report their PA experiments and keep a PA diary. Different ideas for PA were also provided on the project website.	Graded tasks<br>Instruction of how to perform a behaviour<br>Behavioral experiment<br>Action planning<br>Self-monitoring of behavior<br>Monitoring of emotional consequences<br>Verbal persuasion about capability<br>Framing/Reframing	Knowledge	PA experiment and PA diary sheets	
Session 4: Setting SMART-goals	1) Students learn about setting SMART goals, 2) Students set personally meaningful PA goals for themselves and create action plans, 3) Students monitor the achievement of their PA goals	This session started with filling a mid-intervention questionnaire (T2). After this, students discussed their PA experiment home assignments with a class mate, followed by a summarizing discussion as a whole group. Next, a 15 min interactive mini lecture on SMART-goals regarding PA was held. Students were encouraged to individually or with a class mate set PA goals and action plans for next week. The group facilitator provided students with assistance in making their goals SMART. The home assignment of the week was to execute the action plans and monitor the execution by keeping a PA diary.	Review behavior goal(s)<br>Feedback on behavior<br>Discrepancy between current behavior and goal<br>Instruction of how to perform a behaviour<br>Goal setting (behavior)<br>Action planning	Behavioral self-regulation<br>Autonomous motivation<br>Self-efficacy	Powerpoint presentation:<br>SMART PA goals discussion points<br>Individual work sheets for making SMART PA goals and action plans	Face-to-face in class<br>Individual home assignment sheets<br>Project website provided a summary of SMART goals

			Social support (unspecified)<br>Self-monitoring of behaviour<br>Monitoring of emotional consequences<br>Behavioral practice/rehearsal<br>Graded tasks<br>Verbal persuasion about capability			
Session 5: Reviewing and resetting goals	1) Students review their PA goals and receive feedback, 2) Students evaluate the facilitators and barriers of achieving their PA goals, 3) Students learn coping planning skills to prepare for setbacks and plan for strategies to overcome barriers of being physically active	This session started with the students reviewing their last week's PA goal achievement with a class mate and individually setting new goals for the following week with the help of the IV facilitator and class mates. SMART goals were revised during a brief group conversation. Next, students conducted a small group task where they were provided with written cases about youth with different barriers for being physically active. The groups' task was to provide their case with as efficient coping plans for these situations as possible. After working in small groups, the cases and solutions were discussed with the whole class. Finally, students were asked to individually fill in a coping planning sheet combining different barriers for PA with the most functional coping strategies (see Armitage). This week's home assignment was to execute the revised PA goal.	Feedback on behaviour<br>Discrepancy between current behavior and goal<br>Review behavior goal(s)<br>Goal setting (behavior)<br>Action planning<br>Social support (unspecified)<br>Self-monitoring of behaviour<br>Monitoring of emotional consequences	Behavioral self-regulation<br>Self-efficacy<br>Autonomous motivation	Revised SMART goal work sheets<br>Barriers for PA case example sheets<br>Coping planning work sheets	Face-to-face in class<br>Individual home assignment sheets

			Problem solving<br>Behavioral practice/rehearsal<br>Graded tasks<br>Prompts/cues<br>Verbal persuasion about capability<br>Restructuring the physical environment<br>Identification of self as role model			
Session 6: Finish line	1) Students understand what constitutes physical fitness and learn about PA recommendations, 2) Students get an idea of their current level of physical fitness (endurance, strength, mobility), 3) Students learn how to avoid PA related injuries, 4) Students feel competent to maintain a physically active lifestyle	The final session started with a short group revision of last weeks' PA goals and learnings. This was followed by a playful knowledge quiz on 1) areas of physical fitness & ways to improve them and 2) PA & SB recommendations for adolescents. The quiz was conducted in small groups. After the quiz an interactive mini lecture was held on safe ways to improve one's physical fitness and avoid PA related injuries. After this, a 15 min activity break was held. This included playful testing of different areas of students' physical fitness (strength, mobility, aerobic fitness). The final activity of the session was a summarizing group discussion of the main messages and learnings of the intervention. At the end of the meeting student were handed a leaflet with different types of PA goals and exercise ideas related to the different areas of physical fitness. Students were encouraged to	Review behavior goal(s)<br>Feedback on behaviour<br>Discrepancy between current behavior and goal<br>Social support (unspecified)<br>Behavioral practice/rehearsal<br>Graded tasks<br>Instruction of how to perform a behaviour<br>Demonstration of the behavior	Knowledge (also SB)<br>Behavioral self-regulation<br>Autonomous motivation (also SB)<br>Outcome expectations (also SB)<br>Self-efficacy (also SB)	Powerpoint presentation: Instructions for the quiz,<br>Discussion points on PA related injuries<br>Discussion points on main learnings of the intervention<br>Physical fitness goal exercise sheets	Face-to-face in class<br>Individual home assignment sheets

		select the goals that they found personally most meaningful.	Goal setting (behavior)<br>Action planning<br>Verbal persuasion about capability<br>Information about social and environmental / emotional / health consequences<br>Salience of consequences			
Personal booster contact 1	1) Provide students with an opportunity to reflect on their PA after the intervention, 2) Provide individual instruction and support for students in ways of being physically active, 3) Set new PA goals if the students want to	A month after the last intervention session, the facilitator called all students who had given their approval for booster contacts. The phone call was semi-structured to cover the following topics: 1) Review of how the students have felt about PA after the intervention and how motivated they have been in continuing PA, 2) Asking students about their concerns related to PA or setting PA goals, 3) Informing the students about the intervention project's FB-page. Within these frames, the discussion was focused according to the students' own interests. If students were not reached, they were contacted via email where the same points were covered.	Review behavior goal(s)<br>Feedback on behaviour<br>Social support (unspecified)<br>Instruction of how to perform a behaviour<br>Goal setting (behavior)<br>Action planning<br>Problem solving<br>Verbal persuasion about capability<br>Information about social and	Outcome expectations<br>Autonomous motivation<br>Self-efficacy<br>Behavioral self-regulation	Call / email script	1-on-1 via telephone or if participant not reached, via email.

			environmental consequences<br>Information about health consequences<br>Information about emotional consequences<br>Monitoring of emotional consequences			
Personal booster contact 2	Provide students with knowledge on how to build effective PA habits	Two months after the intervention student were contacted again via e-mail. The facilitator thanked the students for participating in the intervention and provided them with ideas on how to build PA habits by using prompts and cues and setting graded tasks for oneself.	Instruction of how to perform a behaviour<br>Prompts/cues<br>Habit formation	Behavioral self-regulation<br>Autonomous motivation		1-on-1 via email
Social media boosters	Revise and boost the learnings from the intervention sessions	On the project FB-page students were provided with printable PA diary sheets, and SMART goal setting sheets. Students were prompted to visit the project website. Students were introduced to alternative ways to monitor PA & arrange social support for being physically active by using social media apps (HeiaHeia). Students were provided with different types of easy and fun ideas for PA (as it was summer, many of the suggested activities could be done outdoors). Students were prompted to set PA related cues to their home environment.	Self-monitoring of behaviour<br>Goal setting (behavior)<br>Action planning<br>Social support (unspecified)<br>Restructuring the physical environment	Behavioral self-regulation<br>Knowledge<br>Self-efficacy<br>Autonomous motivation		Project FB-page updates and discussion among students and facilitator

			Instruction of how to perform a behaviour<br>Behavioral experiment<br>Prompts/cues			

1. There were some differences between the calls with different students depending on what they wanted to discuss.
