# Supplementary material for: Randomised controlled feasibility study of a school-based multi-level intervention to increase physical activity and decrease sedentary behaviour among vocational school students
Source: Int J Behav Nutr Phys Act. 2017 Mar 21;14:37. doi: 10.1186/s12966-017-0484-0 (PMC5361824; doi:10.1186/s12966-017-0484-0)
Supplement: Supplementary file 3 — Specific content of teacher intervention module. (PDF 330 kb) [file 12966_2017_484_MOESM3_ESM.pdf]

Additional Table 2. Specific content of teacher intervention module.

Intervention Session	Aims	Activity description	Key Behaviour Change Techniques	Mediator (theory-based construct)	Materials	Mode of delivery
Workshop I	Teachers:<br>- learn how uninterrupted sitting affects the function of the body and brain<br>- perceive that vocational school teachers and students have positive attitudes towards sitting reduction<br>- acknowledge/ understand the benefits of physical activity to learning, physical health, mood and work climate<br>- practice different strategies to reduce students sitting (activity breaks, active learning methods, light physical equipment)<br>- feel self-confident to implement strategies to reduce students sitting<br>- learn to plan sitting reduction and develop skills on how to motivate student participation<br>- learn how to monitor implementation	In a group formation activity, participants and facilitators were introduced to each other. After this, the facilitator gave a presentation about research results regarding consequences of uninterrupted sitting on physical health, mood and learning. The positive role of physical activity on health, mood and learning was discussed. Teachers were shown how much students on average sit during the school day.<br>To boost motivation for and acceptance of the intervention, results from a pre-study web survey about vocational school teachers' beliefs and attitudes towards increasing physical activity and decreasing sitting at school were shared. A discussion ensued and after this, teachers were prompted to practice different activity break movements and to use light physical equipment. Pedagogical tips on how to motivate students to participate in sitting breaks were discussed. Teachers were asked to set a goal and make action plans for sitting reduction for the next two weeks using a timetable format, where they wrote down their selected sitting reduction strategies and ascribed these to different student groups/classes for the planned implementation (the next two weeks). Teachers were asked to record implementation by circling strategies after each implementation by student group.	5.1/5.6/5.3 Information about health / emotional / social & environmental consequences<br>13.2 Framing/re-framing<br>4.1 Instruction on how to perform the behavior<br>6.1 Demonstration of the behavior<br>8.1 Behavioral practice/rehearsal<br>15.1 Verbal persuasion about capability<br>1.1 Goal setting (behaviour)<br>1.4 Action planning<br>6.3 Information about others' approval<br>5.2 Salience of consequences<br>8.7 Graded tasks	Knowledge<br><br>Autonomous motivation<br><br>Outcome expectations<br><br>Social norms<br><br>Self-efficacy<br><br>Environmental opportunity<br><br>Behavioral self-regulation<br><br>Skills<br><br>Positive group climate	Folders including:<br>a) information about consequences of uninterrupted sitting<br>b) strategies to reduce sitting and practical tips to motivate students<br>c) instructions for basic movements with light PA equipment, and<br>d) powerpoint-slides demonstrating activity breaks.	Face to face meeting in group setting

Between workshops	Teachers:<br>- monitor implementation<br>- review their goals and goal achievement<br>- increase self-efficacy to reduce student sitting<br>- identify barriers in implementation and strategies to overcome the barriers	Teachers received E-mails asking them: 1) Whether they had set a goal for themselves regarding students' sitting reduction. If no goal was set, they were asked to report reasons, 2) To report back on how many times and which sitting reduction strategies they had tried and how they felt about implementing them; 3) To report back on perceived barriers for implementation and on the other hand, strategies they felt had worked well; 4) To report back on student reactions to sitting reduction; 5) To reflect on what would be useful to discuss with other teachers/facilitator in the intervention workshop II (e.g. new sitting reduction strategies, sharing experiences, strategies to motivate students, self-regulation strategies).<br><br>Via email, each teacher received tailored messages that included: 1) compliments for responding and participating; 2) positive feedback about the sitting reduction strategies implemented; 3) practical tips for solving specific barriers, and; 4) encouragement to continue sitting reduction.	4.4 Behavioural experiments<br>3 Social support<br>2.2 Feedback on behaviour<br>2.3 Self-monitoring of behaviour<br>2.4 Self-monitoring of the outcomes of behaviour<br>15.1 Verbal persuasion about capability<br>1.2 Problem solving (in individual feedback)<br>1.5 Review behaviour goals<br>1.6 Discrepancy between current behaviour and goal	Autonomous motivation<br><br>Self-efficacy<br><br>Social support<br><br>Behavioral self-regulation		Personal e-mail
Workshop II	Teachers :<br>- reflect on experiences on sitting reduction<br>- identify facilitators and barriers to reduce students´ sitting<br>- reflect on experienced influences on sitting reduction in students´ and class environment<br>- learn problem solving skills in order to solve problems	Facilitator encouraged group discussions on: 1) experiences of sitting reduction; 2) perceived students reactions to sitting reduction, and; 3) types of co-operation with colleagues and facilitators that could support students' sitting reduction.<br>Barriers reported in E-mails were used as cases, in small groups, with the aim of generating collaborative problem solving discussions.<br>Facilitator gave a short presentation on how to use BCTs (goal setting, action planning, and monitoring) in sitting reduction, along with	3 Social support<br>1.5 Review behaviour goals<br>2.2 Feedback on behaviour<br>15.3 Focus on past success<br>2.3 Self-monitoring the behavior<br>2.4 Self-monitoring the	Autonomous motivation<br><br>Outcome expectations<br><br>Social norms<br><br>Social support<br><br>Self-efficacy	Teachers received a worksheet for coping planning exercise and action planning timetable sheets for the next four weeks. Posters to prompt students' sitting reduction were	Face to face meeting in group setting

	encountered in implementation and create personal plans to solve these in future.<br>- learn to use environmental cues to prompt sitting reduction<br>- learn how to modify goals and make new action plans for sitting reduction	explanations of how these are known to help people reach their goals. Based on their self-monitoring records, teachers shared their goal accomplishments with the group. Then they were prompted to independently reflect on reasons for/barriers associated with goal achievement difficulties and ways of overcoming these barriers.<br>Teachers were prompted to make concrete plans for sitting reduction for the next four weeks and were asked to monitor implementation. Teachers were instructed on how to use environmental cues (e.g. posters, light physical activity equipment in class, timers, students and colleagues) as reminders. These cues were introduced as ways of circumventing forgetfulness (self-regulation issues associated with memory and attention). Teachers once more prompted to reflect on the benefits and reasons why sitting reduction is important. Preferences for ways of follow up contact were asked (e.g. phone calls, e-mails, face-to-face-meetings).<br>Teachers received feedback on goal implementation and were complimented for participation.	outcomes of behaviour<br>1.6 Discrepancy between current behaviour and goal<br><br>1.2 Problem solving<br>7.1 Prompts/cues<br>1.5 Review behaviour goals<br>1.4 Action planning<br><br>5.1/5.6/5.3 Information about health/emotional/ social & environmental consequences<br><br>15.1 Verbal persuasion about the capability	Behavioural self-regulation<br><br>Automaticity<br><br>Skills	delivered to teachers (for classroom walls).<br>Teachers also received new sitting reduction strategies instructions (“active learning methods”).	
Light Physical activity equipment provided for classrooms	Students and teachers receive increased opportunities for reducing sitting during the school day.<br><br>Students and teachers receive increased salient cues prompting physical activity and sitting reduction.	Teachers were given the opportunity to select different light PA equipment to their classrooms to activate classes (gymnastic balls, kettle bells, pilates cushions, gymnastic sticks, balance boards). Equipment was stored in a school storage room where teachers could go and pick them for chosen classes.	30. Restructuring of the physical environment	Environmental opportunity		
