# Supplementary material for: Randomised controlled feasibility study of a school-based multi-level intervention to increase physical activity and decrease sedentary behaviour among vocational school students
Source: Int J Behav Nutr Phys Act. 2017 Mar 21;14:37. doi: 10.1186/s12966-017-0484-0 (PMC5361824; doi:10.1186/s12966-017-0484-0)
Supplement: Supplementary file 4 — Supplementary material. (PDF 202 kb) [file 12966_2017_484_MOESM4_ESM.pdf]

VIRKEYS

VIRKEYS-harjoituksista on ERITYISESTI seuraavia hyötyjä:

- Piristävät mieltä
- Parantavat keskittymis- ja oppimiskykyä
- Tekevät kehosta virkeämmän
- Vähentävät stressiä

Tästä löydät VIRKEYTTÄ tuovia harjoituksia.

WordPress.org

VIDEOT:

- tuolijumppa video engalnniksi (kesto 3:27)
- tuolijumppa video (kesto 1:49)
- lämmittelyharjoitus (kesto 3:00)
- ilmakitara (kesto 3:38)

YKSITTÄISET LIIKKEET (PP-slidet)

- virkeys_kylkien taivutus ja selän rullaus
- virkeys_nyrkkeily_ja_varpaille nousu

LEAVE A REPLY

Your email address will not be published. Required fields are marked *

Name *

Email *

Website

Comment

Supplemental file: Sample page from the Let's Move It teacher intervention website. Translations:

Upper bar: FLEXIBILITY – RELAXATION – ENGERGY – STRENGTH – INCREASING INTERACTION – SIMPLE INSTRUCTIONS FOR PA EQUIPMENT – SIMPLE ACTIVE CLASSROOM LEARNING STRATEGIES – ENVIRONMENTAL CUES
The text lists possible benefits from the exercises and provides links to guided videos (e.g. activity break on a chair, air guitar break) and PowerPoint slides presenting exercises to incorporate in teaching.
